# Solid-Liquid Interdiffusion (SLID) Bonding of p-Type Skutterudite Thermoelectric Material Using Al-Ni Interlayers

**DOI:** 10.3390/ma11122483

**Published:** 2018-12-06

**Authors:** Katarzyna Placha, Richard S. Tuley, Milena Salvo, Valentina Casalegno, Kevin Simpson

**Affiliations:** 1 European Thermodynamics Ltd., 8 Priory Business Park, Leicester LE8 0RX, UK; richard.tuley@etdyn.com (R.S.T.); kevin@etdyn.com (K.S.); 2 Politecnico di Torino, Department of Applied Science and Technology, Corso Duca degli Abruzzi, 10129 Turin, Italy; milena.salvo@polito.it (M.S.); valentina.casalegno@polito.it (V.C.)

**Keywords:** solid-liquid interdiffusion (SLID) bonding, transient-liquid phase bonding (TLPB), skutterudite, high-temperature thermoelectric material, joining

## Abstract

Over the past few years, significant progress towards implementation of environmentally sustainable and cost-effective thermoelectric power generation has been made. However, the reliability and high-temperature stability challenges of incorporating thermoelectric materials into modules still represent a key bottleneck. Here, we demonstrate an implementation of the Solid-Liquid Interdiffusion technique used for bonding *Mm*_y_(Fe,Co)_4_Sb_12_ p-type thermoelectric material to metallic interconnect using a novel aluminium–nickel multi-layered system. It was found that the diffusion reaction-controlled process leads to the formation of two distinct intermetallic compounds (IMCs), Al_3_Ni and Al_3_Ni_2_, with a theoretical melting point higher than the initial bonding temperature. Different manufacturing parameters have also been investigated and their influence on electrical, mechanical and microstructural features of bonded components are reported here. The resulting electrical contact resistances and apparent shear strengths for components with residual aluminium were measured to be (2.8 ± 0.4) × 10^−5^ Ω∙cm^2^ and 5.1 ± 0.5 MPa and with aluminium completely transformed into Al_3_Ni and Al_3_Ni_2_ IMCs were (4.8 ± 0.3) × 10^−5^ Ω∙cm^2^ and 4.5 ± 0.5 MPa respectively. The behaviour and microstructural changes in the joining material have been evaluated through isothermal annealing at hot-leg working temperature to investigate the stability and evolution of the contact.

## 1. Introduction

Skutterudite materials with excellent high-temperature thermoelectric performance and reasonably good mechanical properties are being extensively investigated for power generation use, including automotive waste heat recovery [1,2] and deep space energy generation [3]. However, a major limitation to fully realizing the potential of skutterudites and many other medium-high temperature range thermoelectric materials is the lack of reliable, low-cost assembly technology for robust thermoelectric generators (TEGs). The conventional thermoelectric device manufacturing comprises of labour-intensive methodology including soldering, brazing and adhesive bonding [4] which are limited by the thermal stability (as the braze melting temperature is normally its re-melting temperature) and the extensive growth of reaction layers at the thermoelectric–interconnect interfaces, causing mechanical or electrical deterioration of the entire assembly. In the conventional skutterudite devices, metal interconnects are joined to the metallized semiconductor elements to provide permanent interconnection either by the mechanical (i.e., using compression springs [5]) or chemical (i.e., solid-state diffusion bonding [6,7] or brazing [8]) methods of joining. Designing a suitable metallization and bonding technique for the skutterudite family of compounds is particularly difficult with the complex reactivity of their individual components, since antimony (Sb) can react with most of the transition metals to form brittle antimonide intermetallic compounds (IMCs). Among the many different metallization and interconnects reported, pure elements including those of transition and refractory metals [9,10], nickel-based alloys [11,12] and metal transition silicides [13] are the most commonly explored in a variety of skutterudite-based thermoelectric systems. Single transition metals are proven to not be a suitable metallization for skutterudites as they usually suffer from the intensive thermal stresses induced during the high-temperature service including a volumetric change, associated with the phase transformation at the skutterudite–metallization interface or components thermal expansion coefficient (CTE) mismatch. Although the recently reported Fe-Ni low-expansion alloys exhibit exceptionally good electrical contact resistance of ~0.4 µΩ∙cm^2^ on (Ce_1−z_Nd_z_)_y_ Fe_4−x_Co_x_Sb_12_ p-type material, a higher cost two-step consolidation process can be needed due to the low density of Fe-Ni powders sintered at relatively low temperatures (~85 % of the theoretical value) [13]. Consequently, there is an urgent need for joining processes that could maximize thermoelectric module performance and are transferable to low-cost, high-volume manufacturing.

One promising interconnect technology for high-temperature microelectronic packaging is solid–liquid interdiffusion (SLID) bonding [14], also called transient liquid-phase bonding (TLPB) [15], and diffusion brazing [16]. This technique was developed in the 1970s by Duvall, et al. [17] and constitutes a suitable alternative to high-temperature furnace brazing operations while being commonly considered as the best alternative for solder bonding e.g., for wafer/chip stacked bonding for advanced micro-electro-mechanical systems (MEMS) [18,19] and in sensors manufacturing [20]. The SLID bonding technique is based on binary interlayer systems comprising a high-melting-point material, *T_M HIGH_*, and a low-melting-point material, *T_M LOW_*, which is heated to its molten state, resulting in the formation of intermetallic compounds (IMCs) through a diffusion-reaction mechanism. SLID bonding has been commonly explored using Sn and In as *T_M LOW_* filler systems [21,22,23,24,25] with several advantages over standard solder interconnections such as outstanding thermal stability due to increased re-melting temperature and power handling capability with current densities exceeding the capability of solders [26]. However, SLID technology faces many limitations before industrial implementation due to having a relatively slow and time-consuming diffusion controlled reaction along with inherent Kirkendall voiding and shrink holes created within the bonding material [18]. While, Cu-Sn and Ni-Sn bilayers have been found to form robust IMCs with relatively high melting points, i.e. *T_M_* (Cu_3_Sn) = 676 °C and *T_M_* (Ni_3_Sn_4_) = 794 °C, the possible excess of residual Sn within a joint reduces its attractiveness in high-temperature electronic packaging. To that end, development of new joining materials with high-temperature chemical stabilities and significant oxidation resistances that can be utilized in thermoelectric modules manufacturing are essentially needed.

In the present work, we assess Al-Ni diffusion couples as a potential joining material for high-temperature thermoelectric modules assembly application. Among various intermetallic compounds, Al-Ni IMCs are associated with unique properties such as high thermal stability, tremendous oxidation and corrosion resistance along with excellent performance in creep strength [27]. The aluminium-nickel bi-layer system has already been classified as a particularly suitable structural energetic material used in reactive, multilayer brazing foils due to its exceptional exothermic properties [28,29]. Encouraged by the remarkable properties of Al-Ni IMCs and the recent findings on flux-less solid-liquid interdiffusion (SLID) bonding technique in thermoelectric manufacturing [30,31], a new joining technique utilizing the aluminium–nickel system has been developed and is reported here.

## 2. Materials and Methods

Polycrystalline *Mm*_y_(Fe,Co)_4_Sb_12_ (*Mm*—Mischmetal, i.e., 50 at.% Ce, 25 at.% La) p-type thermoelectric material was synthesized by placing commercial pre-alloyed powder (99.995% purity) inside a graphite die and then sealed with graphite punches. To uniformly distribute heat and forces along sintered material, a graphite sheet (Erodex, Halesowen, UK) was placed between the graphite punch and thermoelectric material. An in-situ synthesis was carried out in Spark Plasma Sintering (SPS) apparatus (FCT HPD25, Rauenstein, Frankenblick, Germany) by applying uniaxial pressure of 50 MPa at the peak temperature of 600 °C for 5 min. The process was performed under a vacuum with heating and cooling rates of 50 °C/min and as-resulted bulk material was 98% dense as determined by the Archimedes principle. Afterwards, the thermoelectric material was ground to 3-mm thick discs using a 320-grit diamond wheel and was cleaned using isopropanol and ultrasonic bath.

Subsequently, 0.3-mm thick copper plate along with as-sintered thermoelectric material were subjected to an electroless nickel plating process with a phosphorous content of 10 wt.% resulting in the nickel–phosphorous Ni(P) coating layer. Both substrates required different surface pre-treatments prior to the electroless nickel plating process due to the poor adherence of the Ni(P) layer on the un-treated skutterudite surface and the presence of a native oxide layer on copper substrates. The thermoelectric material used an electrolytic nickel Wood’s strike comprising NiSO_4_·6H_2_O and 12M HCl electrolyte solution to deposit a seed layer on the surface of the thermoelectric material by passing a current with a density of 5 A/dm^2^ for 2 min. Copper plate was subjected to native oxides removal process including acid cleaning, chemical etching, pre-activation dipping in 5% H_2_SO_4_, and Pd-activation process. Afterwards, both the copper plate and thermoelectric disc were immersed in an electroless nickel plating bath comprising of 45 wt.% NiSO_4_·6H_2_O, 25 wt.% NaPO_2_H_2_·H_2_O and 1 wt.% NH_3_·H_2_O. The bath temperature was kept at 95 °C during the deposition process. As-prepared thermoelectric material was cut to 2.5 × 2.5 × 3 mm rectangular-shaped legs using a diamond cut-off wheel (M1D15, Struers, Ballerup, Denmark) and soaked in acetone to remove any organic residues. A standard scotch tape test was performed on an as-coated thermoelectric surface to evaluate the adhesion quality according to ASTM D3359 standard [32].

**Figure 1:**
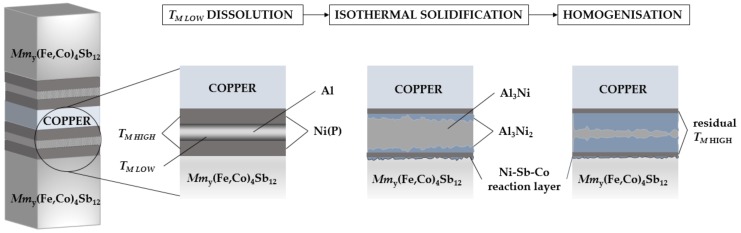
A schematic illustration of the Solid-Liquid Interdiffusion (SLID) bonding process demonstrating the interlayer configuration and joining mechanism when specimens where joined at 660 °C for 4.6 min (described as “*T_M LOW_* dissolution” and “isothermal solidification”) and when isothermally aged at 450 °C for 96 h in Ar (shown as “homogenization”).

A 17 µm-thick Al foil (Goodfellow, Cambridge, UK, 99.0% purity) was incorporated between the Ni(P) coatings on the thermoelectric pellet and the copper plate and supportive uniaxial pressure of ~0.5 MPa was applied. To provide mechanical stability within brazing jig, two symmetrical joints were fabricated by placing a copper plate between two thermoelectric pellets at the same time, as presented in Figure 1. Afterwards, the brazing jig was placed inside a tube furnace (Carbolite, Hope Valley, UK) and the assembly was joined at 585 °C and 660 °C in Ar (4 L/min) with a heating rate of 7 °C/min. All joined assemblies were dwelled at the peak temperature for 4.6 min and afterwards the furnace was switched off and cooled down (at approximately 3 °C/min cooling rate) until the samples reached an ambient temperature. The behaviour and microstructural changes in the joining material were evaluated through isothermal annealing at 450 °C for 48 h and 96 h in flowing argon to investigate the stability and evolution of the joint.

For the microstructural observation, specimens were mounted in conductive resin, mechanically ground with abrasive papers and polished using 1/4 µm diamond suspension. The chemical composition of the reaction products was characterized by high-resolution field emission gun scanning electron microscope (FEGSEM, 1530VP Zeiss GmbH, Oberkochen, Germany) with 80 mm^2^ energy dispersive X-Ray spectroscopy detector (Oxford Instruments, High Wycombe, UK) and analyzed by Aztec EDS/EBSD microanalysis software.

The electrical contact resistance (*R_C_*) of contacting interfaces was periodically measured during the isothermal ageing process using in-house developed four-point probe resistance measurement. The set-up uses a Keithley 2400 (Keithley Instruments, Inc., Cleveland, OH, US) and DPP205 probe positioner (Cascade Microtech, Inc., Beaverton, OR, USA) to measure the voltage drop across two probes as a function of applied short current pulses. The electrical contact resistance (*R_C_*) is given by:

(1) $$R_{C}=\left(R_{1}-R_{0}\right)\cdot A$$

where $R_{1}$, $R_{0}$ are measured resistance at the metallic electrode and the thermoelectric material, respectively, and A is a cross-sectional area of this interface. At least three measurements were made for each specimen prepared at different joining conditions.

**Figure 2:**
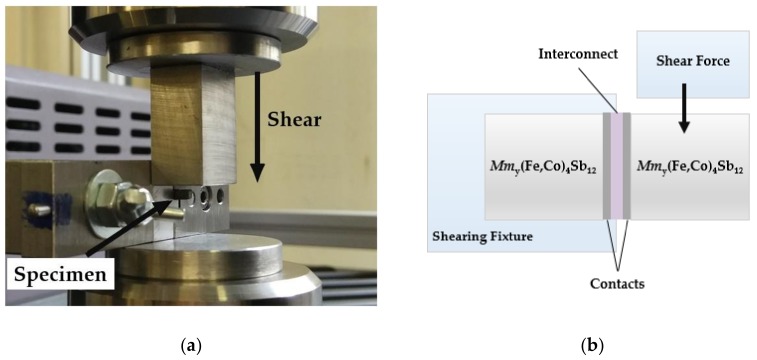
Configuration used for mechanical strength evaluation showing: (**a**) Photograph of sample clamped in the shear test fixture and (**b**) schematic illustration of the specimen and shear test configuration adapted from ASTM D905 standard [24]; the thermoelectric leg length is 3 mm, the copper Ni(P) interconnect is 0.3 mm thick and the contact thickness are ~30 µm. The shearing blade makes contact with the thermoelectric material ≤ 0.3mm from the contact.

The mechanical strength of joints was assessed using specimens with the cross-sectional size of 2.5 mm × 2.5 mm by measuring apparent shear strength using MTS Criterion 43 (MTS Systems Corporation, Eden Prairie, MN, USA) at room temperature. The shear configuration was adapted according to ASTM D905 standard [33] which is designed to expose the assembly to direct contact with a shearing blade at the thermoelectric/joint interface, as depicted in Figure 2a,b. A shear load was applied by moving the blade perpendicularly to the longitudinal axis of the specimen with a speed of 0.2 mm/min. The apparent shear strength was calculated by dividing the maximum force applied by the joining area of at least two specimens of different joining conditions.

## 3. Results and Discussion

### 3.1. Evolution of Microstructure Morphologies

Off-the-shelf *Mm*_y_(Fe,Co)_4_Sb_12_ p-type thermoelectric material (with z*T*_AVG_ = 0.2 measured between 30 °C and 450 °C) was used in this study, with thermoelectric performance significantly lower in comparison to similar compositions previously reported [34]. Commercially available material was used as it is available to be reproduced in the high volume needed for joining trials and this bonding technique is expected to be transferable to other similar skutterudite material compositions. In order to test the feasibility of solid–liquid interdiffusion bonding, Ni(P) coating on p-type skutterudite and copper interconnect was introduced. Phosphorous content in the coating was 18 at.% which, as reported in the literature [35], results in the mixture of amorphous and microcrystalline microstructure. The as-deposited 14 µm-thick Ni(P) layer shows good adherence to the thermoelectric material (4B-level according to ASTM D3359 standard [32]) and is commonly used as a diffusion barrier on copper substrates in semiconductors manufacturing. It has been reported, that due to the lack of high-temperature thermal stability of skutterudites and metal interconnects, implementing an additional diffusion barrier is needed [9,36]. Fundamental research on high-temperature stability between CoSb_3_-based thermoelectric material and Ni interconnect conducted by Chen, et al. [37], revealed extensive growth of reaction layers, i.e., (Co,Ni)Sb and Ni_5_Sb_2_ phases at the skutterudite–nickel interface. In spite of the above-mentioned issues arising from the Ni/CoSb_3_ high-temperature instabilities, no diffusion barrier was implemented as it is beyond the scope of this paper. In this research, the bonding process is designed so that layers of Ni(P)-Al-Ni(P) at the temperature of molten aluminium (*T_M LOW_*) undergo solid–liquid interdiffusion reaction leading to the formation of intermetallic compounds (IMCs) through a diffusion-reaction mechanism (Figure 1). Based on the phase diagram [38], there are five thermodynamically stable intermetallic compounds existing in the Al-Ni binary system but Al_3_Ni and Al_3_Ni_2_ phases are the only two expected to be found in the reactive diffusion zone in both solid–solid and solid–liquid, nickel-aluminium interfaces [39,40]. To achieve the subject of this study for maintaining a single Al_3_Ni_2_ phase between two Ni layers, we calculated, based on the following equation, the initial thickness of Ni needed for 17 µm-thick Al foil:

(2) $$\frac{t_{Ni}}{t_{Al}}=\frac{2M_{Ni}/\rho_{Ni}}{3M_{Al}/\rho_{Al}}$$

where *t* are the thicknesses of both nickel and aluminium layers, *M* are the atomic weights and *ρ* are elemental densities, i.e., 8.9 g/cm^3^ and 2.7 g/cm^3^ of Ni and Al, respectively. However, deviations from the theoretical model were expected as Ni(P) instead of pure nickel was used in this study. According to Equation (2), a symmetrical joint consisting of two 3.75 µm-thick Ni(P) layers and 17 µm-thick Al foil (Figure 1) should be enough to maintain a single Al_3_Ni_2_ phase. While it would be advantageous to completely consume the nickel layer to avoid any subsequent reaction with the skutterudite material, initial experiments indicated that an excess of nickel is required, as any excess aluminium can react with the thermoelectric to form the wider band gap [41] aluminium antimonide, increasing the contact resistance. Moreover, elemental nickel is also consumed by the formation of intermetallic compounds (IMCs) at the skutterudite–Ni(P) interface and its consumption at the interconnection side caused by the significant Cu solubility in Ni [42] at all concentration ranges. Therefore, thicker than calculated Ni(P) layers were deposited.

**Figure 3:**
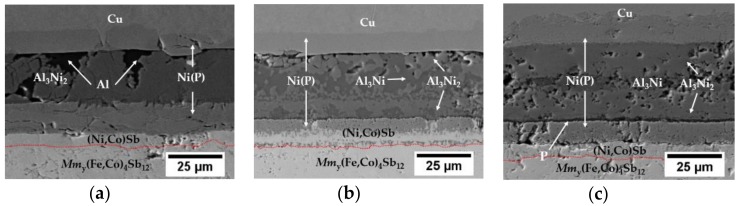
Cross-sectional microstructure of the skutterudite–interconnect interfaces bonded at (**a**) 585 °C; (**b**) 660 °C and (**c**) 660 °C and isothermally aged at 450 °C for 96 h, under flowing Ar, with the Co-Ni-Sb reaction layer marked in red. The high magnification image of Ni(P)–Al_3_Ni_2_ interface can be found in the Supplementary Materials (Figure S1).

Based on the cross-sections of the joints, a 6 µm-thick Co-Ni-Sb reaction layer at the Ni(P)–skutterudite interface can be observed at different bonding conditions (Figure 3a,b) and stays unchanged after isothermal ageing at 450 °C for 96 h (Figure 3c). Based on EDS analysis, the reaction layer is composed of Co—44 at.% Ni—52 at.% Sb which is consistent with results of Ref. [37]. The joining process was performed at two different temperatures, i.e., 585 °C (≤ T_M LOW_) and 660 °C (=T_M LOW_) and the resulting joints’ microstructure can be seen in Figure 3a,b. It is clear that joining at 585 °C is not sufficient for complete phase transformation, as some residual aluminium at the joined interface can be found, and the only formed IMC is Al_3_Ni_2_ (40 at.% Ni and 60 at.% Al) as identified by EDS analysis (Figure 3a). It is believed, that during the heating of the assembly, connected metallic interfaces form a solid solution until its saturation and, as expected, the formation of IMCs through the solid-state diffusion process occurs slower compared to that of the solid–liquid kinetic.

A complete transformation of aluminium filler is observed at a joining temperature of 660 °C as two distinct IMCs, i.e., Al_3_Ni2 (38 at.% Ni and 62 at.% Al) near the Ni(P) coating and Al_3_Ni (24 at.% Ni and 76 at.% Al) in the centre line can be found (Figure 3b), which is consistent with other studies performed on non-thermoelectric, aluminium–nickel diffusion couples [39,43].

A kinetic model was used to explain Al_3_Ni_2_ IMCs growth behaviour and to determine the process heating profile. Considering the joint design based on two nickel-based layers to be contacted with 17 µm-thick aluminium foil, the transition time was calculated based on the formation of Al_3_Ni_2_ at the expense of 7.5 µm of Ni(P) deposit. The thickness of the resulting bonding layer was calculated from the following equation:

(3) $$t_{Al_{3}Ni_{2}}=\frac{M_{Al_{3}Ni_{2}}}{\rho_{Al_{3}Ni_{2}}}\times \frac{t_{Al}\rho_{Al}}{3M_{Al}}$$

where *t* are the thicknesses of both aluminium and the resulting Al_3_Ni_2_ IMCs layer, *M* are the atomic weights and *ρ* are the elemental densities, i.e., 4.7 g/cm^3^ Al_3_Ni_2_ phase. According to Equations (2) and (3), from the consumption of 7.5 µm Ni (P) from both exposed joined surfaces and 17 µm-thick Al foil, a final joint with a 24 µm-thick Al_3_Ni_2_ layer is expected. As shown in Figure 3b, the 28.6 µm-thick joint is thicker than its theoretical estimation which might be induced by the formation of a two-phase bonding region (Al_3_Ni + Al_3_Ni_2_). The parabolic equation based on Fick’s diffusion law was used to determine the bonding conditions as follows:

(4) $$X\left(t,T\right)=k\times t^{n}$$

where *X* is the average thickness of the intermetallic compound, *t* is the joining process time, *T* is the bonding temperature, k is the constant rate, and n is a time exponent. In addition, the temperature dependence of reaction rate k can be expressed by the following Arrhenius relationship:

(5) $$k=k_{0}exp\left(-\frac{Q}{RT}\right)$$

where $k_{0}$ is the frequency factor, *R* is the Boltzmann constant, and *Q* is the activation energy for the growth of the designed Al_3_Ni_2_ phase. According to Tumminello, et al. [44], the two empirical parameters attributed to Al_3_Ni_2_ phase growth, k and n are 8.5 × 10^−^^8^ m/s and 0.844, respectively. Based on Equations (4) and (5), complete transition to the Al_3_Ni_2_ phase can be achieved within 4.6 min, which was used in the experiment. One of the attributes of the SLID bonding process is that both solid–liquid and solid–solid diffusion mechanism play the key roles in isothermal solidification, which makes a process more time consuming than standard soldering [45]. However, due to the high diffusivity of solid nickel in molten aluminium [46], a combination of Al-Ni diffusion couple is a good choice, making the joining process as fast as 4.6 min. Additionally, the resulting IMCs, Al_3_Ni and Al_3_Ni_2_ are characterized with higher than initial re-melting temperature with values of 854 °C and 1133 °C, respectively [38].

In order to investigate the microstructural evolution of the fully transformed contact, isothermal ageing on the assembly joined at 660 °C was performed and the cross-sectional view of that microstructure can be seen in Figure 3c. Isothermal ageing was proven to promote homogenisation within the bond line as a reduction of the Al_3_Ni phase in favour of growing Al_3_Ni_2_ was observed. It is believed that during the homogenisation process, excess nickel from Ni(P) is causing unstable Al_3_Ni phase continual conversion into Al_3_Ni_2_ IMCs through grain boundary diffusion, which may influence the joint’s thermal stability, as the Al_3_Ni_2_ phase has a higher melting point than Al_3_Ni IMC. The residual phosphorous was accumulated at the Al_3_Ni_2_ / Ni(P) interface as a result of Ni depletion from the Ni-P coating due to the formation of desirable Al_3_Ni_2_ IMCs. The high magnification image of the Ni(P)–Al_3_Ni_2_ interface can be found in the Supplementary Materials (Figure S1). Additionally, Kirkendall voids are found within the two-phase region (Al_3_Ni + Al_3_Ni_2_), representing a surface fraction of 4.4%, although some of these are believed to be induced by pulling out the brittle intermetallic grains during the polishing process and might not affect the joining process itself.

### 3.2. Electrical Contact Resistance (R_C_) Evaluation

**Figure 4:**
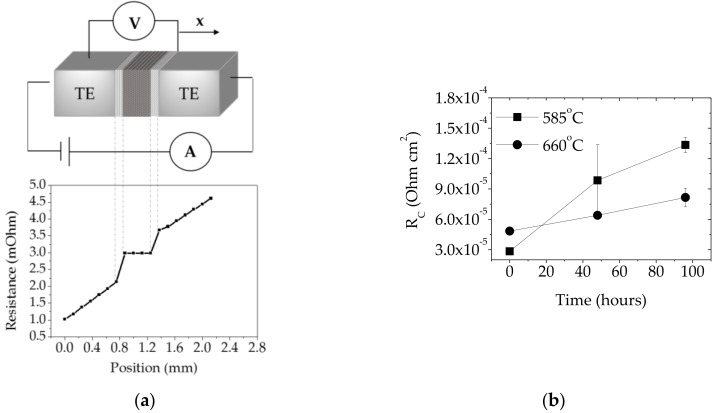
(**a**) Schematic illustration of four-point probe resistance set-up equipped with a scanning probe moving across the surface (x) where the contact resistance (*R_c_*) is defined as the ratio of voltage drop across the contact to the current pulse applied to a pair of contacts; the graph below shows *R_c_* measurement of the skutterudite specimen bonded at 660 °C for 4.6 min and (**b**) *R_c_* evolution of metal interconnect–*Mm*_y_(Fe,Co)_4_Sb_12_ p-type contacts as a function of isothermal ageing time. Note: As-joined contacts are denoted as ‘0 hours’ specimens.

According to Ref. [47], in order to maintain >80% thermoelectric material efficiency in the working device, the electrical contact resistance (*R_c_*) at the thermoelectric–metal contacts needs to be at least less than 30% of the total thermoelectric leg resistance (assuming no thermal contact resistances). By considering one thermoelectric pellet dimension of 2.5 × 2.5 × 3 mm and the electrical resistivity of *Mm*_y_(Fe,Co)_4_Sb_12_ p-type thermoelectric material of 7.59 μΩ∙m (measured by four-point probe resistivity at room temperature), the electrical contact resistance should be < 6.8 × 10^−5^ Ω∙cm^2^ in order to achieve the high module performance promised by the material measurements. The contact resistance (*R_c_*) of assemblies joined at 585 °C and 660 °C was measured to be (2.8 ± 0.4) ×∙10^−5^ Ω∙cm^2^ and (4.8 ± 0.3) × 10^−5^ Ω∙cm^2^ respectively, which contribute approximately 12% and 21% to the total *Mm*_y_(Fe,Co)_4_Sb_12_ p-type thermoelectric pellet resistivity. Moreover, the electrical resistivity of thermoelectric leg was measured using the same technique and showed negligible changes in the material electrical performance after the SLID bonding process. The evolution of the electrical performance during isothermal ageing at 450 °C in flowing Ar, as depicted in Figure 4c, shows that high-temperature degradation leads to the *R_c_* increase by 370% and 68% within 96 h for assemblies joined at 585 °C and 660 °C respectively, which is likely caused by the Al-Ni phase transformation within the joint and a partial Ni(P) delamination from the skutterudite material as observed in SEM images (Figure 3c).

### 3.3. Mechanical Strength Evaluation

**Figure 5:**
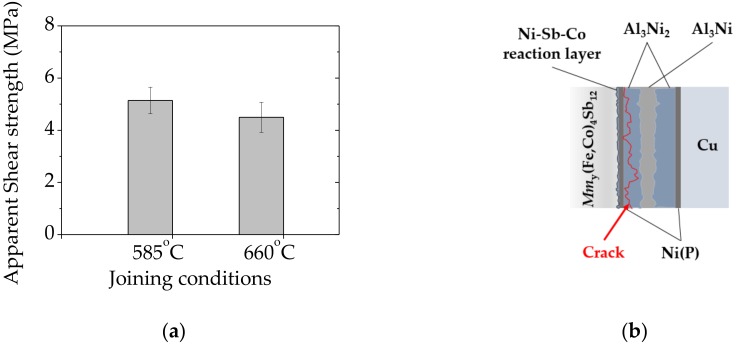
Mechanical performance of bonded specimens: (**a**) Apparent shear strength of interconnect–*Mm*_y_(Fe,Co)_4_Sb_12_ interfaces formed at different joining temperature and (**b**) the schematic illustration with fracture mode of sheared assembly marked in red.

The mechanical strength of bonded specimens was evaluated by measuring apparent shear strength at room temperature adapted to the ASTM D905 standard [33]. As shown in Figure 5a, the apparent shear strength of 5.1 ± 0.5 MPa and 4.5 ± 0.5 MPa was achieved for specimens joined at 585 °C (≤ *T_M LOW_*) and 660 °C (= *T_M LOW_*) respectively. Taking into account that the mechanical properties of skutterudite–metallic interconnects are rarely reported in the literature, and the mechanical strength depends on several variables, i.e., measurement set-up configuration, it is challenging to quantitatively compare to other high-temperature thermoelectric junctions previously reported. Nonetheless, according to Ref. [48], the maximum bonding strength of 13.2 MPa was achieved in low-temperature (Pb, Sn) Te/Cu layers bonded by solid–liquid interdiffusion (SLID) using In-Ag system, suggesting that low mechanical reliability of IMCs-based joints might cause failure in long-term operations, thus requiring further improvement.

**Figure 6:**
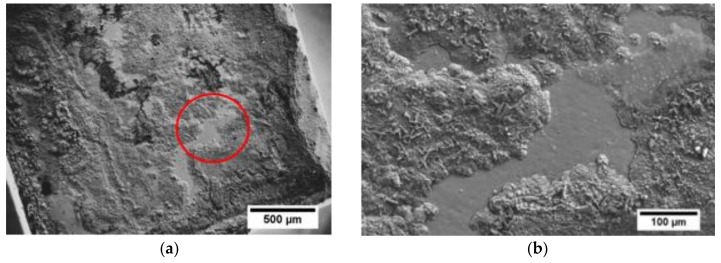

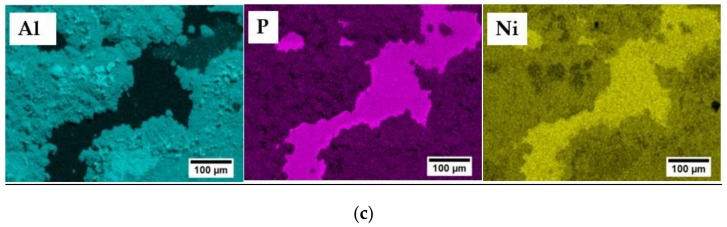
(**a**) Top-view of fractured *Mm*_y_(Fe,Co)_4_Sb_12_ surface after apparent shear strength testing; (**b**) red circle presents the enlarged area shown in (**a**), along with elemental mapping of Al, P and Ni obtained by EDS analysis (**c**).

Scanning electron microscope along with EDS analysis was performed to observe the fractured interface and to understand the possible failure mode occurring within contacts, as presented in Figure 6a,b. The fracture seems to propagate in the mixed mode, along or very close to the Ni(P)–Al_3_Ni_2_ intermetallic interface and within the joining area (as schematically shown in Figure 5b) as the three distinct regions of Ni(P), Al_3_Ni_2_ and Al_3_Ni can be detected by the EDS elemental mapping images.

## 4. Conclusions

It has been shown that Solid-Liquid Interdiffusion (SLID) is a possible technique for bonding metallic interconnects with *Mm*_y_(Fe,Co)_4_Sb_12_ p-type thermoelectric material using aluminium–nickel multi-layered system. Bonding parameters were found to create contacts with a final microstructure falling in the two-phase region comprised of Al_3_Ni and Al_3_Ni_2_ intermetallic compounds. High-temperature reliability and microstructural changes in the joining material have been evaluated in terms of isothermal ageing at 450 °C in flowing Ar, showing that the homogenisation process leads to the reduction of the Al_3_Ni phase in favour of growing Al_3_Ni_2_. In addition, it can be observed that avoiding residual aluminium in the joint by using a higher process temperature is advantageous for improved stability. Although joined components keep their integrity during the high-temperature isothermal ageing, further work is needed to test long term thermal stability including analysis of the potential influence of CTE mismatch of the constituent elements and thermal cycling of prototype modules. Moreover, as the formation of the (Ni, Co) Sb reaction layer at the metal interconnect–skutterudite interface cannot be avoided, implementation of an additional diffusion barrier is still needed.

## Supplementary Materials

The following are available online at http://www.mdpi.com/1996-1944/11/12/2483/s1, Figure S1: (**a**) Cross-sectional microstructure of the skutterudite–interconnect interfaces bonded at 660 °C and isothermally aged at 450 °C for 96 h, under flowing Ar; (**b**) red circle presents the enlarged area shown in (**a**) with a high magnification Scanning electron microscope (SEM) image of the Ni(P)–Al_3_Ni_2_ interface.
