# Supplementary material for: Solid-Liquid Interdiffusion (SLID) Bonding of p-Type Skutterudite Thermoelectric Material Using Al-Ni Interlayers
Source: Materials (Basel). 2018 Dec 6;11(12):2483. doi: 10.3390/ma11122483 (PMC6317193; doi:10.3390/ma11122483)
Supplement: Supplementary file 1 [file materials-11-02483-s001.pdf]

materials

MDPI

Supplementary

Solid-Liquid Interdiffusion (SLID) Bonding of p-Type Skutterudite Thermoelectric Material Using Al-Ni Interlayers

Katarzyna Placha [1,2,*], Richard S. Tuley [1], Milena Salvo [2], Valentina Casalegno [2] and Kevin Simpson [1]

[1] European Thermodynamics Ltd., 8 Priory Business Park, Leicester, LE8 0RX, UK; richard.tuley@etdyn.com (R.S.T.); kevin@etdyn.com (K.S.)

[2] Politecnico di Torino, Department of Applied Science and Technology, Corso Duca degli Abruzzi, 10129, Turin, Italy; milena.salvo@polito.it (M.S.); valentina.casalegno@polito.it (V.C.)

* Correspondence: katarzyna@etdyn.com; Tel.: +44-7922-810-492

Received: 30 October 2018; Accepted: 4 December 2018; Published: 6 December 2018

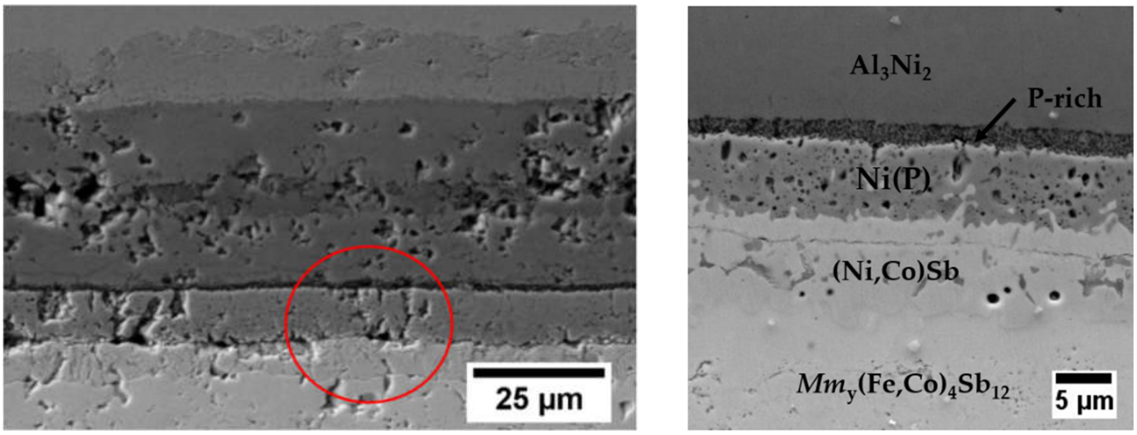

(a) **(b)**

Figure S1. (**a**) Cross-sectional microstructure of the skutterudite–interconnect interfaces bonded at 660 °C and isothermally aged at 450 °C for 96 h, under flowing Ar; (**b**) red circle presents the enlarged area shown in (**a**) with a high magnification Scanning electron microscope (SEM) image of the Ni(P)–Al_3Ni_2 interface.
